# Supplementary material for: DeepCME: A deep learning framework for computing solution statistics of the chemical master equation
Source: PLoS Comput Biol. 2021 Dec 8;17(12):e1009623. doi: 10.1371/journal.pcbi.1009623 (PMC8687598; doi:10.1371/journal.pcbi.1009623)
Supplement: Attachment — Submitted filename: Gupta_review.pdf [file pcbi.1009623.s001.pdf]

1 Reviewer comments

This work introduces a novel algorithmic approach to estimating Chemical Master Equation (CME) solutions by using simulated trajectories to train a Deep Neural Net (DNN). The key advantages of this approach are: (1) the method generally estimates desired quantities (e.g., some summary statistics or other function of the distribution $p(x, t)$, which is the full solution of the CME), more efficiently than by direct sampling; (2) perhaps even more significantly, the method also outputs parameter sensitivities.

The work looks to me to represent a significant advance in an area of high interest. I would think the method would be of high interest outside of biological applications, as well (indeed a historical home for CME methodology papers is Journal of Chemical Physics), though the authors do not discuss applications outside biology/biochemistry. The results are generally very convincing and the paper looks to be novel, important, and rigorous. However I note below several questions about the methodology that should either be addressed or clarified.

I found the paper to be written in a way that seemed targeted to readership well familiar with both stochastic process theory and deep learning. Some of my suggestions for improvement below involve making the conceptual underpinnings of their method more accessible to a broad (but mathematically/computationally literate) readership.

1.1 Comments on methodology

Re: efficiency gain of DeepCME as compared to direct sampling. Efficiency gain (in obtaining both the function estimates and parameter sensitivities) seems to be the major advantage of DeepCME–especially, as the authors' results show, in DeepCME's favorable scaling of CPU time with increased system size. However, I had some questions related to how this efficiency gain was measured.

1. To my understanding, the authors used Anderson's next reaction method for the training batch of simulated trajectories in DeepCME, whereas the direct simulation ("benchmark") in Figs. 2-5 uses SSA. Can the authors comment on the reason for this discrepancy? I wondered whether one could fairly assess the efficiency gain (as represented by CPU time, panel B) when different algorithms are used for the simulations in the two approaches. Especially if Anderson's method is more efficient than SSA.

Why not use the same simulation algorithm for both DeepCME and direct simulation?

2. Also related to CPU time and panel B, Figs. 2-5: it was not clear how exactly the stopping points were chosen for SSA and DeepCME. To my understanding, the authors used 1000 trajectories (SSA) and 10000 steps (DeepCME). But the CPU time comparisons would seem more meaningful if both methods were run to some common stopping point, say to reaching within some defined tolerance of the (known) exact result, at least for Figs. 2-3.

3. Is the CPU time to generate the training batch of simulated trajectories included in the CPU time plotted for DeepCME? It would seem that for fair comparison of total CPU, it should be. However then it also seems that the total of BPA and SSA should be used for comparison on the direct sampling side, since, to my understanding, the BPA is needed separately from SSA to obtain parameter sensitivities, whereas DeepCME outputs parameter sensitivities together with the function estimates at once. To summarize this point, I wondered whether the results in panels B give a potential end-user an appropriate idea of the efficiency gain of DeepCME? Or are these figures intended to highight the different scaling with system size, rather than direct comparison of efficiency? In any case, the way CPU time is computed should be clarified.

Other points:

1. There are a few cases in Figures 2-3 where the SSA function estimates are surprisingly far from the exact result. Or more precisely where the exact result is actually outside the estimated 95% confidence interval from SSA. Though surprising to me, perhaps the explanation is simply that 1000 SSA trajectories is too few. However, this calls into question the usefulness of SSA as a benchmark in Figs.4-5 (where there is no exact result and thus SSA should in principle be providing the necessary benchmark). I recommend the authors confirm that their SSA simulations will converge to the exact result with increased sampling for one of the studied systems in Figs. 2-3.

1.2 Comments on clarity and communication

1. The authors provide relatively little conceptual explanation about how and why the method works. Adding more such explanation could improve understanding of the method for a broad readership. For example:

   (a) The highly interesting question underlying efficiency gain of DeepCME is: how is it that the DeepCME method is able to effectively get more information out of few trajectories as compared to direct sampling/estimation? I wished the authors would spend more time

addressing this question. The only relevant sentences were in the introduction, where they state: "However, we find that the number of paths to achieve DNN training generally is lower than by direct use of Monte-Carlo estimator combined with SSA; accuracy is achieved through the generalization properties of DNNs rather than through approximation admissible sets of initial densities." Could the authors elaborate on this somewhere in the paper, especially since a broad readership may not be familiar with the generalization properties of DNNs? Related questions are: Could an alternative mathematical formulation to estimating CME solutions (e.g., one based on forward Kolmogorov?) achieve similar efficiency gains, or is there something special about their particular formulation and choice of policy map?

(b) The quantity $\mathcal{V}_k(t, X(t))$ seems key to the method. This is the function that is learned by the DNN, which allows estimation of the quantities of interest $E(g(X(t))$, as well as the parameter sensitivities. Yet it is never explained what this function is/means, conceptually. I think perhaps the components $\Delta_k V_g$ could be interpretable as: "For a given state of the system x, the effect of a difference of a single event of firing reaction k prior to time t on the expectation of g at time T." I hoped the authors could provide some such conceptual definition and then further elaborate on why that function (and equation (21)) is key to the method. (This also relates to the previous point).

(c) Figure 1 seems not particularly illuminating in terms of communicating how the DNN works. In particular, the inputs are confusing. It would helpful to show schematically where the simulated training data fits in. (To my understanding, after studying the paper, the time t is essentially user-defined (e.g., they looked at uniform intervals on $[0, T]$), the vector x is the state of the system at time t (copy numbers of each species) as obtained from a training simulation, and the λ eigenvalues and phase shifts are unknown a priori but trained by the algorithm.) I'm not quite sure how the batch of trajectories is input. Are states at different time points of a trajectory mixed with the ones of other trajectories? It would be helpful to improve the figure, or at least the caption, to clarify how all of these inputs are obtained. Alternatively, if it would not be appropriate to add this information into Fig. 1, perhaps a second schematic could be added somewhere that represents the whole pipeline, from training simulations to "deliverables" (the function estimates and parameter sensitivities).

(d) What does it mean that sensitivities w.r.t. all parameters are obtained in one shot? Does it mean that they do not have to vary any of the parameters in the training simulations?

2. I find the title of the paper and abstract slightly misleading, because of the phrase "solving the CME". The method does not approximate the

full distribution $p(x,t)$, but rather some (presumably small number of) user-defined summary statistics or other function of the distribution, for example, the first and second moments. Perhaps it's just an issue of opinion/semantics, but it would be clearer if at least the abstract was more precise about their meaning of solving the CME.
