# Supplementary material for: DeepCME: A deep learning framework for computing solution statistics of the chemical master equation
Source: PLoS Comput Biol. 2021 Dec 8;17(12):e1009623. doi: 10.1371/journal.pcbi.1009623 (PMC8687598; doi:10.1371/journal.pcbi.1009623)
Supplement: Attachment — Submitted filename: ReviewerResponse.pdf [file pcbi.1009623.s002.pdf]

Response Letter

DeepCME: A deep learning framework for computing solution statistics of the Chemical Master Equation

Ankit Gupta*[1], Christoph Schwab†[2], and Mustafa Khammash‡[1]

[1]Department of Biosystems Science and Engineering, ETH Zürich, Mattenstrasse 26, 4058 Basel, Switzerland.

[2]Seminar für Angewandte Mathematik, ETH Zürich, 8092 Zürich, Switzerland.

September 6, 2021

We thank the reviewers of *PLOS Computational Biology* for going through our paper and making many important remarks. We have revised our manuscript accordingly. We describe and comment below all the changes which have been included in the resubmission in detail.

The parts of the paper that have been added/modified appear in blue to allow the reviewers to locate these parts easily. We first mention all the major changes that have been made to the manuscript and second, we provide a detailed response to each of the reviewers' comments.

Major Changes

1. The title and the abstract have been modified to clearly mention that our method is designed to estimate expectations based on the CME solution, rather than obtain the CME solution directly.

2. In the previous version of the manuscript, the benchmark function estimates were generated with Gillespie's SSA even though our method requires simulations generated by Anderson's modified next reaction method (mNRM). To correct for this discrepancy, we have redone all the examples with mNRM and consequently, all the figures have been updated. The principal findings reported in our original submission remain unchanged.

*ankit.gupta@bsse.ethz.ch

†christoph.schwab@sam.math.ethz.ch

‡mustafa.khammash@bsse.ethz.ch

Response to Reviewer 1

1. *This work introduces a novel algorithmic approach to estimating Chemical Master Equation (CME) solutions by using simulated trajectories to train a Deep Neural Net (DNN). The key advantages of this approach are: (1) the method generally estimates desired quantities (e.g., some summary statistics or other function of the distribution p(x; t), which is the full solution of the CME), more efficiently than by direct sampling; (2) perhaps even more significantly the method also outputs parameter sensitivities. The work looks to me to represent a significant advance in an area of high interest. I would think the method would be of high interest outside of biological applications, as well (indeed a historical home for CME methodology papers is Journal of Chemical Physics), though the authors do not discuss applications outside biology/biochemistry. The results are generally very convincing and the paper looks to be novel, important, and rigorous.*

   Answer: We thank the reviewer for these kind words about our paper.

2. *However I note below several questions about the methodology that should either be addressed or clarified. I found the paper to be written in a way that seemed targeted to readership well familiar with both stochastic process theory and deep learning. Some of my suggestions for improvement below involve making the conceptual underpinnings of their method more accessible to a broad (but mathematically/computationally literate) readership.*

   Answer: We thank the reviewer for insisting on these clarifications. This helped us improve the readability of our paper. In the revision, we have tried to address all these issues.

3. *Re: efficiency gain of DeepCME as compared to direct sampling. Efficiency gain (in obtaining both the function estimates and parameter sensitivities) seems to be the major advantage of DeepCME - especially, as the authors' results show, in DeepCME's favorable scaling of CPU time with increased system size. However, I had some questions related to how this efficiency gain was measured.*

   1. To my understanding, the authors used Anderson's next reaction method for the training batch of simulated trajectories in DeepCME, whereas the direct simulation ("benchmark") in Figs. 2-5 uses SSA. Can the authors comment on the reason for this discrepancy? I wondered whether one could fairly assess the efficiency gain (as represented by CPU time, panel B) when different algorithms are used for the simulations in the two approaches. Especially if Anderson's method is more efficient than SSA.

   Why not use the same simulation algorithm for both DeepCME and direct simulation?

   Answer: This is a valid point. For our method we generate simulated trajectories with Anderson's modified next reaction method (mNRM) as it is based on Kurtz's random time-change which lies at the heart of our method. We used Gillespie's SSA

as the benchmark because it is more commonly used for stochastic simulations than the next reaction network, even though the latter is more efficient.

In order to correct for this discrepancy we now use Anderson's mNRM for direct simulations as well. The figures are updated accordingly.

4. *2. Also related to CPU time and panel B, Figs. 2-5: it was not clear how exactly the stopping points were chosen for SSA and DeepCME. To my understanding, the authors used 1000 trajectories (SSA) and 10000 steps (DeepCME). But the CPU time comparisons would seem more meaningful if both methods were run to some common stopping point, say to reaching within some defined tolerance of the (known) exact result, at least for Figs. 2-3.*

   Answer: We understand the reviewer's point but having a common stopping point for both methods is difficult as these methods are very different. In particular for DeepCME, which is implemented with TensorFlow, a fixed batch of training trajectories is generated a priori and then a fixed number of training steps are performed. For reasons related to computational efficiency, it is not possible to check the stopping criterion after each training step and/or increase the size of training trajectories if convergence fails. Moreover for simulation-based schemes (SSA and BPA) it is unclear how additional trajectories should be allocated to both the methods when the stopping criterion is not reached. Moreover for many of our examples, it can take an exorbitant amount of computational time to reach a predefined tolerance criterion with simulation-based schemes.

   We realise that using 1000 trajectories for simulation-based schemes and 10000 training steps for DeepCME may seem like arbitrary choices. However the main point of this comparison is to provide the user with an idea of how DeepCME performs in comparison to standard Monte Carlo estimators (for function estimates and sensitivities) with a modest number of samples.

5. *3. Is the CPU time to generate the training batch of simulated trajectories included in the CPU time plotted for DeepCME? It would seem that for fair comparison of total CPU, it should be. However then it also seems that the total of BPA and SSA should be used for comparison on the direct sampling side, since, to my understanding, the BPA is needed separately from SSA to obtain parameter sensitivities, whereas DeepCME outputs parameter sensitivities together with the function estimates at once. To summarize this point, I wondered whether the results in panels B give a potential end-user an appropriate idea of the efficiency gain of DeepCME? Or are these figures intended to highlight the different scaling with system size, rather than direct comparison of efficiency? In any case, the way CPU time is computed should be clarified.*

   Answer: Yes, the CPU time for generating the training batch is included for DeepCME. We make this clear in the revised manuscript (see page 14). The reviewer's understanding is correct - the BPA is needed separately from SSA to obtain parame-

ter sensitivities, whereas DeepCME outputs parameter sensitivities together with the function estimates at once. Therefore for simulation-based approaches the CPU time includes the time needed for both SSA and parameter sensitivities with BPA. We clarify this on page 14 of the revised manuscript and also mention this in the figure captions. Of course now we replace the SSA with the modified Next Reaction Method (mNRM) to address the reviewer's first comment.

6. *Other points: 1. There are a few cases in Figures 2-3 where the SSA function estimates are surprisingly far from the exact result. Or more precisely where the exact result is actually outside the estimated 95% confidence interval from SSA. Though surprising to me, perhaps the explanation is simply that 1000 SSA trajectories is too few. However, this calls into question the usefulness of SSA as a benchmark in Figs.4-5 (where there is no exact result and thus SSA should in principle be providing the necessary benchmark). I recommend the authors confirm that their SSA simulations will converge to the exact result with increased sampling for one of the studied systems in Figs. 2-3*

   Answer: The SSA is an exact method and, as the reviewer pointed out, the reason that the exact result is outside the 95% confidence interval is because the number of trajectories (1000) is not sufficiently large. In the revised manuscript we provide additional simulation-based estimates with 10000 trajectory simulations in examples where the exact result is not available. This confirms empirically the convergence of simulation-based results.

7. *1.2 Comments on clarity and communication 1. The authors provide relatively little conceptual explanation about how and why the method works. Adding more such explanation could improve understanding of the method for a broad readership. For example: (a) The highly interesting question underlying efficiency gain of DeepCME is: how is it that the DeepCME method is able to effectively get more information out of few trajectories as compared to direct sampling/estimation? I wished the authors would spend more time addressing this question. The only relevant sentences were in the introduction, where they state: However, we find that the number of paths to achieve DNN training generally is lower than by direct use of Monte-Carlo estimator combined with SSA; accuracy is achieved through the generalization properties of DNNs rather than through approximation admissible sets of initial densities." Could the authors elaborate on this somewhere in the paper, especially since a broad readership may not be familiar with the generalization properties of DNNs? Related questions are: Could an alternative mathematical formulation to estimating CME solutions (e.g., one based on forward Kolmogorov?) achieve similar efficiency gains, or is there something special about their particular formulation and choice of policy map?*

   Answer: We thank the reviewer for raising these issues. In the revised text, we provide a more detailed explanation (see Remark 3.4 on page 8) on why we think DNN extracts more information out of simulated trajectories in comparison to direct sampling. A complete, mathematical justification of this empirical observation is beyond the scope of the present paper, as it would require significant additional mathematical research.

As this point we cannot address conclusively the reviewer's question about whether an alternative formulation of the CME could achieve similar efficiency gains. However, this is certainly an interesting direction for future research.

8. *(b) The quantity $\mathcal{V}_k(t, X(t))$ seems key to the method. This is the function that is learned by the DNN, which allows estimation of the quantities of interest $\mathbb{E}(g(X(t))$, as well as the parameter sensitivities. Yet it is never explained what this function is/means, conceptually. I think perhaps the components $\Delta_k \mathcal{V}_g$ could be interpretable as: "For a given state of the system x, the effect a difference of a single event of firing reaction k prior to time t on the expectation of g at time T." I hoped the authors could provide some such conceptual definition and then further elaborate on why that function (and equation (21)) is key to the method. (This also relates to the previous point).*

   Answer: We now provide an interpretation of $\mathcal{V}_k(t, X(t))$ and explain its role in our method in greater detail (see Remark 3.4 on page 8).

9. *(c) Figure 1 seems not particularly illuminating in terms of communicating how the DNN works. In particular, the inputs are confusing. It would helpful to show schematically where the simulated training data fits in. (To my understanding, after studying the paper, the time t is essentially user-defined (e.g., they looked at uniform intervals on $[0, T]$), the vector x is the state of the system at time t (copy-numbers of each species) as obtained from a training simulation, and the eigenvalues and phase shifts are unknown a priori but trained by the algorithm.) I'm not quite sure how the batch of trajectories is input. Are states at different time points of a trajectory mixed with the ones of other trajectories? It would be helpful to improve the figure, or at least the caption, to clarify how all of these inputs are obtained. Alternatively, if it would not be appropriate to add this information into Fig. 1, perhaps a second schematic could be added somewhere that represents the whole pipeline, from training simulations to deliverables" (the function estimates and parameter sensitivities).*

   Answer: We understand the confusion and we have revised Section 4 to address this issue. What is important is to note that the DNN only acts like an approximator of the matrix valued map $(t, x) \mapsto \mathcal{V}(t, x)$. The DNN *does not directly take as inputs* the batch of simulated trajectories. This batch of trajectories is employed for training the DNN by passing time-state pairs (t, x) generated by each trajectory (at various times) to the DNN and then using the outputs $\mathcal{V}(t, x)$ to estimate the loss function, as described in Section 4.2. Please see the explanation we have added on page 11.

10. *(d) What does it mean that sensitivities w.r.t. all parameters are obtained in one shot? Does it mean that they do not have to vary any of the parameters in the training simulations?*

    Answer: We have elaborated on that in Section 2.3 in the main paper. Basically it means that from the training data and the trained DNN we can estimate the sensitivities w.r.t. all the parameters without much extra effort, by simply evaluating a Monte

Carlo estimator based on the sensitivity formula mentioned in Section 2.3.

11. *I find the title of the paper and abstract slightly misleading, because of the phrase "solving the CME". The method does not approximate the full distribution p(x; t), but rather some (presumably small number of) user-defined summary statistics or other function of the distribution, for example, the first and second moments. Perhaps it's just an issue of opinion/semantics, but it would be clearer if at least the abstract was more precise about their meaning of solving the CME.*

    Answer: We have modified both the title and the abstract to address this issue.

Response to Reviewer 2

At a high level, the authors are using machine learning techniques (basic feedforward neural networks) to solve for (time dependent) expectations of stochastically modeled reaction networks. I think that this paper has the potential to be very good. It is the rare paper I review in which I think to myself "I wish I had done this!". Here are my main comments/critiques that I would like the authors to consider.

1. *The title claims to solve the chemical master equation (Kolmogorov's forward equation). However, the paper is actually focused on solving for expectations. Now, I agree that a solution to the CME can come from using indicator functions. However, that does not really seem to be a natural application for this method (and there are no examples showing if the method is good for that).*

    Answer: We have revised the title and the abstract to make it clear that our method is able to estimate expectations of the solution of the CME. In future we do hope to improve the method to a point where accurate estimates of the "full" CME solution can be obtained.

2. *A very important part of the paper is Theorem 3.3. More precisely: equation (19), which gives an equality that must hold (almost surely) for every *path*. In my view, the heart of the proof is that m(t) (unnumbered equation between (23) and (24)) is a local martingale. A few more words can be given here (or a reference) proving/saying that $\Delta\widehat{\mathcal{V}}_k(s, X(s))$ is adapted to the proper filtration and $\widetilde{R}_k$ is a local martingale, thus.... Pointing to the proper reference will be helpful to readers not familiar with these things.*

    Answer: We thank the reviewer for this comment. In the previous version we had mentioned the local martingale property of $\widetilde{R}_k$ and provided a reference right after defining $\widetilde{R}_k$ in Section 3. Now we have also added statements on the local martingale property of $m(t)$ and adaptedness of $\Delta\widehat{\mathcal{V}}_k(s, X(s))$.

3. *Page 10. Why was ϕ as given chosen? (this is minor)*

Answer: We explain the choice of ϕ in the paragraph following its definition.

4. *This is my only major critique. I could not figure out exactly what the input to the NN was. This is explained on pages 10 and 11, but I did not understand it. Of course, I would really like to know how a path gets inputed since this is an absolutely key part of the method. I wonder if a simple example would be helpful here (I would suggest the birth death process of section 5.1).*

   Answer: We have revised Section 4 to explain the workflow of DeepCME more clearly. Please see the explanation that we have added on page 11. As we mention in our response to point 9 of reviewer 1, the DNN only acts like an approximator of the matrix valued map $(t, x) \mapsto \mathcal{V}(t, x)$. When a path is simulated the time-state pairs (t, x) are passed to the DNN and the outputs $\mathcal{V}(t, x)$ are used to estimate the loss function, as described in Section 4.2.

5. *Why were the NN's used so small ($L = 2$ and $N_H = 4$)?*

   Answer: We did try many other combinations of L (no. of layers) and N_H (no. of nodes/layer). However we found that with our feedforward architecture for DNN, increasing L and N_H does not improve convergence of the DNN training process or the quality of the estimates. We illustrate this point with the "linear signalling cascade" example in Section 5.2.

6. *The method does not seem viable at this stage for computing sensitivities. Perhaps soften your language to something along the lines of "we note that sensitivities can theoretically be computed as well via these methods" you could then give the reasoning as is. However, you could point out that it sometimes works and sometimes doesn't (since the examples are all over the map), and then point to this as future research.*

   Answer: While discussing the examples we acknowledge that in some cases the estimated parametric sensitivities are not accurate (especially when the network size is large). In the conclusion section we have softened our language regarding sensitivity estimation along the lines suggested by the reviewer.

Response to Reviewer 3

The DeepCME is a novel deep learning framework for numerical solution of the chemical master equation (CME). The authors reformulate the problem of obtaining expectation of specific functions of state space and their sensitives using the Kolmogorov's backward equation (theorem 3.3). Using this reformulation they construct appropriate loss functions to train deep neural networks using a reinforcement framework using relatively small number of stochastic simulations (SSA). They demonstrate the power and utility of the their method using a series of examples. This is a timely, well written and important study. I have the following specific comments:

1. *Could the authors provide some insight the advantage of the specific formulation compared for example to forward equation in solving CME using deep neural networks?*

   Answer: The main reason for using the backward formulation is to derive an almost sure relationship that should be satisfied by each trajectory (Theorem 3.3). This allows our DNN-based method to extract more information from each simulated trajectory as we explain on page 8 of the revised manuscript (see Remark 3.4). It may be possible to construct similar formulations with the forward equation and this in an important direction for future research.

2. *At the beginning of section 5 the author's describe specific choice of a large number of hyper parameters (number of layer's, nodes, etc). Could the authors explore the significant of at least some of these choices in an example on the performance?*

   Answer: The main DNN parameters are L (no. of layers) and N_H (no. of nodes/layer). As we mention in response to reviewer 2 (see point 5), we tried many other combinations of these parameters but they did not change the results significantly. In the revised manuscript, we illustrate this point with the "linear signalling cascade" example in Section 5.2.

3. *The author's show the CPU time required for direct SSA vs DeepCME. Could the author's also make comparison in term's of number of simulations used in the training and how does that change with n and the quality of the results?*

   Answer: In our setup we use a fixed number of simulated trajectories for training which is 100. The comparison requested by the reviewer is difficult to provide because it is hard to find a common criterion (across all values of n) of when the DNN can be considered "trained". Nevertheless to address the reviewer's concern, we apply DeepCME to the "linear signalling cascade" network with $n = 10$ species and show that increasing the number of training trajectories from $M = 100$ to $M = 500$ does improve the accuracy of the estimates (see Section 5.2 for more details).
