# Supplementary material for: DeepCME: A deep learning framework for computing solution statistics of the chemical master equation
Source: PLoS Comput Biol. 2021 Dec 8;17(12):e1009623. doi: 10.1371/journal.pcbi.1009623 (PMC8687598; doi:10.1371/journal.pcbi.1009623)
Supplement: Attachment — Submitted filename: ReviewerResponse2.pdf [file pcbi.1009623.s003.pdf]

Response Letter

DeepCME: A deep learning framework for computing solution statistics of the Chemical Master Equation

Ankit Gupta*[1], Christoph Schwab†[2], and Mustafa Khammash‡[1]

[1]Department of Biosystems Science and Engineering, ETH Zürich, Mattenstrasse 26, 4058 Basel, Switzerland.

[2]Seminar für Angewandte Mathematik, ETH Zürich, 8092 Zürich, Switzerland.

November 1, 2021

We thank the reviewers of *PLOS Computational Biology* for again going through our paper. We have revised our manuscript to address the comments of reviewer 2. The parts of the paper that have been added/modified appear in blue to allow the reviewers to locate these parts easily. We now provide a detailed response to each of Reviewer 2 comments.

Response to Reviewer 2

1. *The manuscript has improved. However, one of my main worries about the manuscript (and apparently this worry was also shared by Reviewer 1) remains unresolved. In particular, the inputs to the NN are still quite unclear to me. Let me be very explicit: I have no idea what is going on with the lambda's and phi's of what is now section 4.1. The authors describe them as "temporal features", but that doesn't mean anything to me. A trajectory is simply a listing of states and times. The authors need to explain how a trajectory is inputed into the NN. If there is a mapping to the lambda's and phi's somehow, then so be it, but please tell us clearly what that mapping is. At this point I would not be able to implement this method as the whole portion on "temporal features" is mysterious.*

   Answer: We think that the confusion stems from the fact that the input to the NN is a time-state pair (t, x) rather than a trajectory. Hence a trajectory is not directly inputed to the NN but the time-state pairs it generates, at a discrete set of time-points, are sequentially inputed to the NN to obtain the corresponding values of the policy map $\mathcal{V}(t, x)$. Based on these policy-map values, the discrepancy in the almost

*ankit.gupta@bsse.ethz.ch
†christoph.schwab@sam.math.ethz.ch
‡mustafa.khammash@bsse.ethz.ch

sure relation provided by Theorem 3.3 is iteratively computed for each trajectory. Computing these discrepancies for a batch of trajectories, the final loss function is evaluated and subsequently minimised by altering Φ (the weights and biases in the NN) to train the policy map. We have clarified this issue of inputs to the NN in the revised Section 4.2. Please note that for a fixed Φ, the NN-encoded policy-map remains the same for each trajectory.

The temporal features for the policy map are selected based on the form of the solution of the backward Kolmogorov's equation for finite Markov chains, and they are akin to "basis functions" in a typical regression approach. These temporal features are parametrised by λ-s which are also adjusted during the training process.

2. *Here are some smaller comments: In the abstract it again says "The goal... estimating solutions of high-dimensional CMEs..." this is not really what it is doing. The next line is more accurate. Maybe merge and fix? (Except the next line says "Arbitrarily chosen functions" which seems strange)*

   Answer: We replaced the phrase "estimating solutions" to "computing solution statistics" as in the title. When we say "arbitrarily chosen functions" we mean that the functions that define the solution statistics can be arbitrary. To avoid confusion we have replaced the phrase "arbitrarily chosen" with "user-defined".

3. *(Minor). Line 63. "Which for" to "for which"*

   Answer: Actually in the context of this sentence "which for" was correct. However we have modified the sentence to make its meaning clearer.

4. *Line 123, this is super small but in the definition of the Q matrix you seem to be assuming that each reaction vector is uniquely associated with a reaction. This is not always the case.*

   Answer: Yes, as mentioned in the footnote just before the definition of this matrix we assume that all stoichiometric vectors are distinct for convenience.

5. *Line 143. "On which our deep learning approach depends on" — one too many "on"s*

   Answer: Thanks for pointing this out. We have removed the extra "on" at the end.

6. *Same line. Which is same → which is the same.*

   Answer: Thanks. We have corrected this.

7. *Line 250. Why is this line here: "The relation between ϕ and its realisation $R(\phi)$ as a map is not one-to-one." I guess it's true: there can be lots of choices of rho's and T's that give R. But is this important (i.e., I'm concerned I'm missing something)?*

   Answer: This line was just to emphasise that the DNN encoded policy map is *over-parametrised* and hence the loss function may have multiple minima. We have added a sentence to mention this.
